# Supplementary material for: Are carbon emissions trading and green financial instruments synergistic? -Comprehensive quantitative research based on content analysis
Source: PLoS One. 2024 Mar 7;19(3):e0298601. doi: 10.1371/journal.pone.0298601 (PMC10919601; doi:10.1371/journal.pone.0298601)
Supplement: S1 Data — (ZIP) [file pone.0298601.s001.zip › Data/地级市Z_F工作报告.zip!/╦─┤¿/└╓╔╜╩╨/└┤╫╘╣1⁄2╓┌║┼í╛┬φ┐╦╩2╛▌═°í┐_2017.docx]

各位代表：

　　现在，我代表乐山市人民政府向大会报告工作，请予审查，并请市政协各位委员提出意见。

　　2016年工作回顾

　　2016年是实施“十三五”规划、全面建成小康社会决胜阶段的开局之年。一年来，我们深入学习贯彻党的十八大、十八届三中、四中、五中、六中全会和习近平总书记系列重要讲话精神，在市委的坚强领导下，在市人大、市政协的监督支持下，坚持“干在实处、走在前列”的工作取向，紧扣“创新投资年”工作主题，积极应对各种困难和风险挑战，千方百计稳增长、促改革、调结构、惠民生、防风险，实现了“十三五”良好开局。全市实现地区生产总值1406.6亿元、增长8.2%;全社会固定资产投资1109.8亿元、增长12.3%;规模以上工业增加值增长9.8%;社会消费品零售总额624.3亿元、增长13.1%;地方一般公共预算收入93.1亿元、增长9%;城镇居民人均可支配收入28583元、增长8.4%，农村居民人均可支配收入12749元、增长9.4%;主要经济指标增速均高于年初预期、高于全省平均水平。

　　一年来，我们重点抓了以下六个方面工作：

　　一是抓投资稳增长。我们坚持把项目作为稳增长调结构补短板的重要抓手，制定重点推进项目挂图作战工作方案和重大项目三年攻坚实施方案，新入库500万元以上项目1149个，23个省列重大项目完成投资163.9亿元，38个挂图作战项目完成投资187.3亿元，分别完成计划任务的132.3%、106.4%。乐沙城际生态大道、仁沐新高速仁井段建成通车，万达广场、峨眉山黄湾小镇建成开放。坚持把稳工业作为稳增长的主体，建立重点困难企业解困清单，开展降低实体经济企业成本行动，工业整体产能发挥率稳定在85%左右，规模企业产销率由年初的94.3%回升至96.9%。城镇化进程持续加快，新增城镇建成区面积4.35平方公里，城镇化率达48.7%。积极拓展市场消费，开展各类促销活动600余场次，电商交易额增长30%，网络零售额增长25%，商品住房销售面积增长30%。

　　二是抓产业强支撑。我们坚持以发展质量和效益为中心，主动调结构、转方式，三次产业结构调整为10.9：57.5：31.6，第三产业比重上升1.5个百分点。把旅游作为服务业的龙头产业、主导产业来培育，成功举办第三届旅博会、2016环中国国际公路自行车赛、峨眉山国际登山节、佛光花海音乐节、申遗20周年纪念活动等大型文旅活动，四川旅博天地创建为国家4A级景区，全市实现旅游综合收入625.7亿元、增长25.3%，接待国内外游客4380万人次、增长11.7%。深入实施服务业“三百工程”，规模以上服务业企业净增10家、达164家，限额以上批发零售和住宿餐饮企业净增19家、达620家。加快工业转型步伐，启动“一总部三基地”建设，硅材料循环经济标准化试点通过国家验收，永祥500兆瓦硅片、峨胜新型干法水泥环保搬迁等项目竣工投产，新兴产业主营业务收入占规模以上工业比重提高到39%。节能减排力度加大，实施减排项目68个，单位地区生产总值能耗预计下降4.66%。大力转变农业发展方式，成功举办首届茶博会，新建成现代特色效益农业标准化基地13万亩，新发展农民专业合作社334个、家庭农场328家，认证“三品一标”农产品89个，完成粮食总产量110.7万吨。

　　三是抓脱贫补短板。我们坚持把脱贫攻坚作为“头等大事”来抓，累计整合投入各类扶贫资金76.7亿元，实现了41个省定贫困村、98个市列贫困村退出，3.5万贫困人口脱贫，脱贫摘帽首战告捷。大力改善贫困地区基础设施条件，竣工彝家新寨住房1750户，完成易地扶贫搬迁9261人，改造建档立卡贫困户危房4919户。创新开展“百企帮百村”活动，市内464户企业(合作社)与469个贫困村结成帮扶对子。探索资产收益、投资收益、理财收益、金融造血扶贫等模式，设立100亿元脱贫攻坚专项基金，发放支农再贷款3.9亿元、扶贫再贷款1.2亿元、扶贫小额信贷1.9亿元。建立教育扶贫救助基金，新建“一村一幼”155所，解决彝区3204名学生“两人一铺”问题，“三县一区”全部执行15年免费教育。全面落实贫困人口医疗救助政策，新建村卫生室161个，建立卫生扶贫救助基金，实现贫困人口县域内住院政策范围费用零支付。强化政策兜底，实现低保线、贫困线“两线合一”，对3679名建档立卡困难群众实施居家和集中救助帮扶。创建市级“四好村”378个。对口援助理塘工作圆满结束，启动对口援助美姑县和市内对口援彝工作。

　　四是抓改革添动力。我们坚持把改革创新贯穿于经济社会各个领域，新出台专项改革方案34项，13项国家部委改革试点、26项省级改革试点取得积极进展，成功申报为国家服务业综合改革试点市。出台推进供给侧结构性改革实施意见，淘汰落后产能企业17户，关闭小煤矿28处。农业农村改革有序推进，土地承包经营权确权登记颁证全面完成，新增土地流转面积3万亩、达56万亩。国资国企改革不断深化，市属国企实现集中统一监管。商事制度改革全面推进，工商登记前置审批事项从206项减少为51项，新登记企业4086户、增长16%。财税金融改革取得实效，4支政府投资基金组建运作，“1+7+N”国有投融资平台积极构建，全市本外币各项存款余额1864.8亿元、贷款余额1311.2亿元，分别较年初增加137.2亿元、72.9亿元。启动实施不动产统一登记制度，颁发不动产权证书9979本。创新驱动深入实施，成立西南旅游众创联盟、乐山中科创新育成中心，新增科技企业孵化器2家、国家检验检测机构1家，专利实施项目新增产值111.2亿元、增长18.5%，高新技术产业实现产值550亿元、增长13.6%。开放合作纵深拓展，组团参加西博会、中外知名企业四川行、川商返乡发展大会等活动，引进到位市外内资560亿元，实际利用外资5500万美元，分别完成年度目标任务的112%、110%。

　　五是抓民生促和谐。我们坚持以人民为中心的发展思想，全面完成“十项民生工程”和22件民生大事，群众生活进一步改善。狠抓就业创业工作，城镇新增就业4.96万人，城镇登记失业率控制在4.1%以内。深入实施全民参保计划，稳步提高低保、优抚、退休人员养老金待遇等标准。优先发展教育事业，市中区、五通桥、夹江等3个区县通过国家级义务教育均衡发展达标验收认定，乐师附小建成投用，新建农村教师周转房451套、农村中小学学生宿舍3万平方米，接收16144名进城务工随迁子女就近免试入学。推进医药卫生体制综合改革，公立医疗机构全面取消药品加成。人口自然增长率为2.67‰。深化百万安居工程建设行动，建成保障性安居工程住房13758套，完成省下任务的179%。加强生态环境保护，综合治理水土流失面积273平方公里，岷江出境断面水质取得突破性改善。沐川、峨边、马边等3个县纳入国家重点生态功能区，大瓦山跻身国家湿地公园，全市森林覆盖率达56.39%。认真落实党政同责、一岗双责、齐抓共管、失职追责，深入开展安全隐患大排查大整治，安全生产形势总体平稳。立体化社会治安防控体系不断完善，群众和信访工作得到加强，社会保持和谐稳定。我市被表彰为全国“六五”普法先进单位，连续四届荣获全国双拥模范城称号。

六是抓效能优环境。我们坚持转变政府职能，扎实推进政府系统“两学一做”学习教育，不断加强政府自身建设，发展环境进一步优化。深入推进简政放权、放管结合、优化服务，公布市政府组成部门权力清单、责任清单，全部取消非行政许可事项。创新“互联网+政务服务”，建成行政权力依法规范公开运行平台和网上办事电子政务大厅。开展横向“并联审批”和纵向“三级联审”，对重大项目实施全程代办服务。开播“嘉廉话”阳光问廉全媒体直播节目，聚焦脱贫攻坚、城乡环境整治等重点工作，公开问责问效问廉。严格执行中央八项规定和省市十项规定，完成公务用车制度改革，实现政府部门派驻监督全覆盖。始终保持反腐高压态势，处置问题线索1341件，立案717件，党纪政纪处分710人。自觉接受市人大及其常委会的法律监督、工作监督和市政协的民主监督，办结市人大代表建议、市政协委员提案440件，满意和基本满意率达100%。

各位代表：回顾过去一年的政府工作，成绩来之不易。这是省委、省政府和市委坚强领导的结果，是市人大、市政协和社会各界监督支持的结果，是全市广大干部群众共同奋斗的结果。在此，我代表市人民政府，向全市各族人民，向驻乐部队指战员、武警消防官兵、政法干警，向各民主党派、工商联、无党派人士、各人民团体和各界朋友表示衷心的感谢，并致以崇高的敬意!

　　在看到成绩的同时，我们也清醒地看到，我市经济社会发展仍面临一些困难和问题，主要表现为：结构调整任务艰巨，主导产业不够明显，服务业比重与全国、全省平均水平相比还有较大差距;经济下行压力依然较大，民间投资信心不足，部分企业生产经营较为困难，稳增长的措施还需进一步加强;税源结构单一，缺乏新的税源增长点，财政收支压力持续加大;保障和改善民生任务依然艰巨，全市还有8.3万名群众处于贫困线下;政府职能转变力度还需加大，公共服务效率需要进一步提高等等。对此，我们高度重视，正在采取积极措施，认真加以解决。

　　2017年工作安排

　　2017年是乐山加快转型发展的重要一年，也是新一届政府的开局之年。市政府工作的总体要求是：全面贯彻党的十八大和十八届三中、四中、五中、六中全会精神，认真落实以习近平同志为核心的党中央治国理政新理念新思想新战略，紧紧围绕“五位一体”总体布局和“四个全面”战略布局，深入实施省委“三大发展战略”，坚持稳中求进工作总基调，以供给侧结构性改革为主线，以“基础设施建设年”为主题，着力项目建设，全力脱贫攻坚，强化改革创新，加快结构调整，不断改善民生，促进全市经济社会平稳较好发展，推动各项工作走在全省前列。主要目标是：地区生产总值增长8%左右，全社会固定资产投资增长11%，规模以上工业增加值增长9.2%，社会消费品零售总额增长12%，地方一般公共预算收入增长8%，城镇居民人均可支配收入增长8%，农村居民人均可支配收入增长9%，全面完成省下达的各项目标任务。我们将重点抓好以下工作：

　　一、突出基础设施推进项目建设

　　围绕“基础设施建设年”主题，持续扩大有效投资，确保全社会固定资产投资完成1200亿元以上。

　　攻坚重大项目。发挥重大项目对经济社会发展的支撑引领和转型升级导向示范作用，抓好294个省市重大项目建设，确保省市重大项目完成投资506亿元。对“一总部三基地”、岷江航电综合开发、峨眉山国际佛教文化旅游目的地综合开发、三江流域生态涵养修复工程、苏稽新区、乐西高速等6个列入全省100个重点推进的项目，细化进度，倒排工期，全力推进，确保取得突破性进展。全面推进挂图作战，继续实行“一个项目、一名领导、一支队伍、一套方案、一抓到底”，强化“六包”责任，力争今年56个挂图作战项目完成投资259亿元。围绕发展定位，大手笔策划包装、生成储备一批支撑力强、带动性大、惠及面广的项目，不断充实、调整、优化项目储备库。

　　聚力重点领域。全面推进“三年交通大会战”，竣工乐自高速乐山城区连接线、成昆铁路成峨段项目，加快建设岷江航电犍为枢纽、成贵铁路、连乐铁路、成昆铁路峨米段、仁沐新高速井沐马段、峨汉高速等项目，开工建设岷江航电老木孔枢纽和龙溪口枢纽、乐西高速、成乐高速扩容等项目，力争完成交通投资200亿元。着力推进地方公路建设，竣工乐峨大道、乐沙大道、井乐快速通道，加快建设省道308改线、乐夹大道、五犍沐快速公路，开工建设眉乐快速干道、省道215线过境公路，打造“半小时经济圈”和“一小时经济圈”。有序推进城市建设项目计划，开工建设致江路桥、绕城高速互通、大佛连接线和通棉路改造等项目，确保嘉瑞大道、瑞祥路等竣工通车，完成城建投资260亿元以上。挖掘民间投资潜力，出台促进民间投资健康发展的实施意见，鼓励民间资本参与公共服务领域建设，提高民间投资比重。

　　破解制约瓶颈。强化要素保障，下深水解决制约项目建设的融资、用地、立项审批等问题，提高项目落地成熟度。做强“1+7+N”国有投融资平台，综合利用政府投资基金、地方债券、PPP、直接上市融资等方式，多渠道筹集项目建设资金。完善土地精准保障机制，积极实施增减挂钩、废弃地复垦等项目，盘活利用存量和低效闲置土地，依法依规做好征地拆迁工作，保障重大项目用地需求。清理规范投资项目报建审批事项，完善投资项目在线审批监管平台，探索企业投资项目承诺制和报建手续先建后验试点，畅通项目审批“绿色通道”，促进立项审批全面提速。

　　二、着力旅游主导做强第三产业

　　把推动服务业发展提速、比重提高、水平提升作为调结构、促转型的主攻方向，大力发展以旅游为龙头的现代服务业，力争服务业增加值占地区生产总值比重提高到33%。

　　发展全域旅游。推动峨眉山、乐山大佛景区扩容提质，加快峨眉山景区基础设施和黄湾小镇后续项目建设，启动乐山大佛景区南大门景观整治、凤洲坝旅游开发项目，积极创建峨眉国家级旅游度假区和乐山大佛省级旅游度假区。抓好港中旅乐山旅游资源整体开发项目，积极打造黑竹沟、千佛岩·东风堰、大瓦山、竹海、美女峰、故宫文物南迁遗址公园等特色旅游景区景点，力争罗城古镇创建为国家4A级景区，加快嘉阳·桫椤湖创国家5A级景区进程。突出历史文化名城保护，加快老城区历史文化保护挖掘，启动中心城区禅意景观廊道、绿心公园绿色生态整治等项目，推进景城一体发展。加大旅游宣传营销，建设国家级旅游行业云计算(大数据)中心，精心办好第四届旅博会、第二届茶博会等活动，力争全年旅游综合收入、接待游客人次分别增长20%、15%。

　　优化服务业态。以建设国家服务业综合改革试点市为契机，积极实施服务业倍增计划，加快建设区域性服务业中心城市，促进服务业发展提质增效升级。积极发展电子商务业，加大与京东、淘宝、唯品会等大型知名电商平台合作力度，推动猪八戒网乐山园区项目落户高新区，加快腾讯旅游实战基地建设，新建乡(村)电商服务站点100个、县级电子商务孵化园3个。积极发展养老健康业，挖掘道地药材、生态养生等优势潜力，创新医养、康养等发展模式，打造养老健康服务业示范区。积极发展科技服务业，打造省级百亿科技服务业重点市。积极发展现代金融业，推进农信社改革，有序发展社区银行、村镇银行、农村资金互助社，加快普惠金融体系建设，争创科技金融结合试点。积极发展现代物流业，改造提升餐饮、住宿、娱乐、商贸等传统服务业，新培育规模以上服务业企业25家。

　　激活城乡消费。打造全国质量强市示范城市、全省放心舒心消费城市，整顿和规范市场秩序，改善提升市场消费环境。积极搭建促销平台，加大与万达、华联、王府井、沃尔玛等大型商场超市合作，开设特产专柜、离境退税店，提升“乐山造”产品知晓度和竞争力。稳定住房消费，支持房地产企业大力开发旅游、康养、金融等地产，满足城镇居民改善型需求和新市民住房需求，促进房地产市场平稳健康发展。深化国家信息消费试点市建设，加快完善信息基础设施，实施“全企入网、全民触网、电商示范”工程，推动信息产品融合创新。积极把握居民消费升级趋势，大力培育发展休闲旅游、教育文体、绿色环保等新兴消费。鼓励城乡居民合理增加创业性、财产性、经营性收入，促进城乡居民收入增长与国民收入增长相协调，增强城乡居民消费能力。

三、打造总部基地推进工业转型

　　围绕“一总部三基地”战略布局，打造“总部+基地”功能链条，推动全市工业集中布局、集聚发展、集成创新。

　　加快园区建设。制定完善“一总部三基地”发展规划，理顺园区管理、建设、运行和利益分享体制机制，编制园区产业指导目录，制定支持产业转移政策，促进全市工业企业向“一总部三基地”有序平稳转移，力争工业集中度提高到75%。支持高新投公司做大做强，加快建设高新区总部大楼、高新广场和商务金融特色街区，推动乐山国家农业科技园区总园落户高新区。全面启动“一总部三基地”道路、管网、给排水等基础设施建设，实现“一年拉框架”目标。加快园区产业化项目建设，抓好新万兴1000万平方米碳纤维地暖陶瓷和二期100吨航空复合材料、润和5万吨高效稀土催化剂、凤生12万吨生活用纸技改等项目，力争“一总部三基地”产值达200亿元以上。

　　调优产业结构。加快发展新材料、光电信息、医药健康、先进装备等新兴产业，抓好和邦10万吨蛋氨酸、永祥5万吨高纯多晶硅和2000兆瓦硅片、新天源1000兆瓦硅片技改扩能等项目，力争新兴产业主营业务收入增长10%，占规模以上工业比重提高1个百分点。大力发展军民融合产业，加快推进16个纳入全省“十三五”军民融合重点项目建设，力争红华实业三期、医用同位素溶液堆、军民两用物流园年内开工。推动传统产业绿色化改造，加快岷江化工公司、吉象木业、长药城区老工业区环保搬迁，抓好福华30万吨离子膜烧碱扩能技改、玖龙65万吨牛卡纸扩能技改、夹江6000万平方米陶瓷生产线技改等项目，完成技改投资250亿元。

　　壮大企业集群。着力构建“亲”“清”新型政商关系，毫不动摇地鼓励、支持、引导民营经济发展，振兴实体经济。开展降低企业成本行动和质量对标提升行动，在减税、降费、降低要素成本上下功夫，全力稳定企业现生产，帮扶一批重点困难企业脱困解困。实施规上企业培育五年倍增计划，力争新增主营业务收入超20亿元企业1家、超10亿元企业2家，新培育规模以上工业企业50家。积极实施科技、电商等中小微企业成长培育计划，加大对种子期、初创期成长型小微企业支持力度，培育壮大中小微企业群体。大力发扬“工匠精神”，切实加强品牌建设，培育一批“百年老店”，增强产品竞争力。

　　四、壮大现代农业促进农民增收

　　始终把解决好“三农”问题作为工作的重中之重，围绕促进农民持续增收这个核心，推进农业供给侧结构性改革，加快发展现代农业，不断提高农业发展质量、效益和竞争力。

　　发展规模经营。抓好“一区六带”建设，积极推进现代农业(林业)重点县、示范县建设，大力发展茶叶、林竹、现代畜牧、蔬菜、中药材等特色产业，抓好土壤地图推广应用，新建现代经济作物产业标准化基地12万亩。大力培育新型农业经营主体和服务主体，推广“公司+基地+合作社+农户”等经营模式，完善利益联结机制，新发展各类新型农业经营主体100个，培育市级重点龙头企业10家、农业科技园区企业5家，农业产业化带动面提高1个百分点。加快农村土地流转，放活农村土地经营权，深化农村承包土地的经营权抵押贷款试点，引导土地经营权流向种养能手和新型经营主体。

　　培育特色品牌。依托茶博会、农博会、农交会等展示展销平台，加大“峨眉山”“小凉山”等区域品牌推介和宣传力度，建成全国峨眉山茶产业知名品牌示范区。大力发展生态、有机农业，完成“三品一标”产品认证25个，新发展有机农产品基地4万亩。积极发展农旅融合特色小镇、休闲农庄、农耕体验基地等休闲农业，大力发展乡村旅游，提高农业全产业链收益。积极推广“互联网+农业”，拓宽特色农产品销售渠道，推动农业产业化创新发展。加强农产品质量和农业投入品质量监管，健全从农田到餐桌的全过程监管体系，确保农产品质量安全。

　　完善基础设施。实施百个水生态项目、百亿水利投入、百公里江河堤防建设“三百水利工程”，新建加固堤防25公里，整治病险水库5座。推进农田水利基本建设，大力实施防洪减灾、安全饮水、渠系灌排等水利工程，新增有效灌面4.5万亩。抓好农村路网、电网、信息网建设，新改建农村公路1040公里，改造升级农村电网2500公里，基本实现广播电视户户通。建成幸福美丽新村200个，完成农村住房改造2380户。

　　五、奋力脱贫攻坚确保连战连捷

　　按照“两不愁、三保障”和“四个好”要求，统筹推进连片扶贫和插花扶贫，实现沐川县精准摘帽，95个省定贫困村、71个市列贫困村精准退出，3.3万贫困人口精准脱贫。

　　实施扶贫专项。持续改善贫困地区基础设施条件，完成易地扶贫搬迁13261人，新建彝家新寨28个。以阻断贫困代际传递为着力点，加快发展民族教育，巩固提升15年免费教育、“一村一幼”“一村一支教”工作，新建农村教师周转房1000套、农村中小学学生宿舍5万平方米，全面消除彝区学生“两人一铺”问题。积极推动落实国家开发银行信用助学贷款工作。加大医疗救助力度，严格落实贫困人口“十免四补助”、先诊疗后结算等政策，防止因病返贫、因病致贫。坚持农村低保应保尽保，同步提高低保线、贫困线“两线合一”标准，高质量实施居家和集中救助帮扶工作。有效整合金融资源，加大扶贫创业担保贷款、易地扶贫搬迁、联保贷款等专项贷款力度，新发放扶贫小额信贷1亿元。

　　开展对口帮扶。深入开展“百企帮百村”，带动发展生态特色产业，增加贫困群众种养业收入。继续开展驻村帮扶，加强“五个一”驻村帮扶管理监督，提升驻村帮扶工作实效。深化“1+3”对口帮扶，重点帮扶美姑县编制旅游发展规划，推动6个扶贫新村建设，加快峨美路、马美路改扩建工程，完成乐山一中第一届“美姑班”招生，同步推进市内三对区县对口帮扶工作。做好中纪委、浙江省和17个省级单位对口帮扶协调服务，确保各类帮扶项目和资金落地落实。

　　强化励志扶贫。持续开展“新家园、新生活、新风尚”系列评选活动，巩固深化“亲情工作法”、彝区“德古”依法调解、礼金聘金“双高治理”，推动形成崇尚科学、遵法守法的良好风气，创建市级“四好村”350个。推动知识下乡，办好农民夜校，实施技能培训和就业促进计划，实现“一人就业、全家脱贫”。加强感恩奋进教育，加大脱贫攻坚先进典型宣传力度，引导群众饮水思源、自力更生，用勤劳双手改变落后面貌、创造幸福生活。

　　六、坚持问题导向深化改革创新

　　以提高经济发展质量和效益为中心，坚持问题导向倒逼改革创新，促进新动能发展壮大、传统动能焕发生机。

　　推进重点改革。坚持“四个有利于”改革导向，认真落实国家和省上改革部署，推动全面深化改革迈出更大步伐，创造出更多可推广、可复制的乐山经验。深化供给侧结构性改革，以煤炭、陶瓷、铁合金、纺织等行业为重点，鼓励企业兼并重组，处置一批“僵尸企业”，关闭小煤矿7家以上。落实国家级旅游业改革创新先行区政策，建立完善旅游发展引导、评价考核、投融资机制，设立旅游文化产业发展基金。深化国资国企改革，加快企业现代法人治理结构建设，加大国有企业经理层市场化选聘力度，推进国有企业整合重组和混合所有制改造，提高核心竞争力和资源配置效率。推进财税体制改革，合理划分财政事权和支出责任。深化商事制度改革，探索注册登记全程电子化，进一步降低创业创新制度性成本。统筹推进教育、文化、司法等领域改革。

鼓励创新创业。深入推进大众创业、万众创新，建立创新创业投资引导基金，培育一批国家级、省级创业园区。强化企业创新主体地位，支持优势企业与高等院校、科研院所组建技术研发平台和产业技术创新联盟，推动产学研用协同创新，科技进步综合水平指数提高到53%。实施高新技术产业倍增计划，力争新培育国家高新技术企业15家，新增创新创业平台载体5个，孵化支持各类创新创业主体或项目100个，推动科技成果、专利转化为现实生产力，完成高新技术产业产值650亿元以上。积极实施“嘉州英才工程”，加快引进和培育一批技能型、实用型、紧缺型人才，为创新创业提供强大人才支撑和智力支持。

　　促进开放合作。坚持“对内靠改革、对外靠开放”，拓展与长三角、泛珠三角、京津冀等经济圈的交流合作，深化与成都平原城市群、成渝经济区的区域合作，以扩大开放带动创新、推动改革、促进发展。围绕优势主导产业、战略性资源开发和产业发展布局调整，组织策划专题招商活动，招高引新、招大引强，促进一批重点招商项目快签约、快建设、快投产，引进到位市外内资600亿元以上。加强外向型企业、园区和出口示范基地建设，探索建立保税物流区、跨国产业园区等专业平台，力争实现进出口总额9亿美元。

　　七、筑牢绿色理念建设生态家园

　　牢固树立“绿水青山就是金山银山”的理念，切实改善生态环境，推进新型城镇化建设，全力打造山水园林宜居城。

　　打造宜居城乡。坚持“绿色打底、山河为骨、文化为魂”，全力推进“一城两新区”建设，新增城镇建成区面积4平方公里，城镇化率提高到50.1%。实施老城区保畅、减容、增绿、控高工程，疏解部分医疗、教育、文体、专业市场等功能，新建停车场11个、新改扩建公厕109个。全面启动苏稽新区大学城、文化馆、体育馆、科技馆、规划馆、博物馆等“一城五馆”及会展中心规划建设，加快冠英新区湿地公园、污水管网等项目建设，竣工冠英污水处理厂。推进智慧城市、海绵城市、地下综合管廊建设，建设市大数据中心，提升城市综合管理水平。统筹推进宜居县城建设试点，加快“四个特色”品牌创建，建成特色小镇13个、特色街区6个、特色村落50个，培育特色产业品牌100个。

　　构建绿色屏障。开展全市主体功能区总体规划中期论证，推进永久基本农田和生态保护红线划定，制定和落实生态保护红线负面清单。完成“一湖五湿地”规划编制，实质性启动峨眉河湿地公园、竹公溪湿地公园、岷江生态湿地公园、三江湿地公园、大渡河湿地公园建设，争取桫椤湖创建为国家级湿地公园。实施“绿秀嘉州”工程，建设一批绿色通道、绿色河道、绿色村庄、绿色园区、绿色城区示范试点，推进新一轮退耕还林还草、天然林保护、水土流失治理等工程，森林覆盖率提高到56.72%。维护生态系统平衡，加强重点生态功能区、自然保护区、生态涵养区保护，推动绿心保护立法，守护好绿水青山、蓝天净土。

　　整治环境污染。打好大气、水、土壤和固体废弃物污染防治攻坚战，健全区域大气污染联防联控机制，抓好工业废气治理，加强城市扬尘、餐饮油烟管控，推进机动车污染治理，落实农作物秸秆禁烧，有效减少雾霾污染，切实改善空气质量。全面推行河长制，加强岷江、青衣江、大渡河等重点流域及其支流水环境防控，加大茫溪河、凌云河等小流域污染治理力度，加快茫溪河补水暨湿地公园项目建设，推进乡镇污水处理设施建设全覆盖。抓好集中式饮用水水源保护区整治，竣工市第五自来水厂。开展土壤污染调查，实施农用地分类管理和建设用地准入管理。加强固体废物污染防治和危险废物监管，确保工业固体废物处置利用率达到95%以上。推动环境保护多元共治、多主体制衡，建立生态补偿机制，严格执行生态环境损害赔偿制度和责任追究制度。

　　八、办好民生实事补齐民生短板

　　围绕保障和改善民生，补齐社会事业发展短板，做好社会托底工作，织密扎牢民生保障网，不断增强人民群众获得感。

　　办好民生实事。深入实施“十项民生工程”，突出办好民生大事。加强就业创业平台建设，打造“乐创汇”“众创联盟”等创业活动品牌，力争城镇新增就业3.8万人以上。落实基本医疗保险、城乡居民大病保险、医保医师、医疗救助等制度，调整提高城乡低保、特困人员供养等标准，推进机关事业单位养老保险制度改革。抓好公租房“租改售”试点和城镇危旧房棚户区改造，棚改全部实现货币安置。积极发展妇女儿童、老龄、慈善事业，完善未成年人社会保护体系。

　　发展社会事业。推进义务教育均衡发展，逐步消除大班额现象，完成3个县国家级义务教育均衡发展达标验收。扩大学前教育资源，新增普惠性学位1000个。抓好乐山一中、市实验中学、实验小学、实验幼儿园等新改扩建项目，推进乐山职业技术学院、乐山一职中、乐山二中整体搬迁，积极筹建书画学院、旅游学院和四川特殊教育职业技术学院，启动大学城基础设施建设。深化医药卫生体制综合改革，完善分级诊疗、医保支付制度改革，加强中医药工作，提升基层医疗服务能力。加快市人民医院国际医疗康养中心、市妇幼保健院整体迁建等项目建设。组织实施一批文化惠民工程，打造一批文化精品力作，举办一批文化走基层主题活动，确保国家公共文化服务体系示范区通过中期验收。落实全民健身国家战略，举办市第七届运动会和市第二届残疾人运动会。开工建设市残疾人综合服务中心。加快推进市殡仪馆迁建项目。开展第三次全国农业普查。继续抓好民族宗教、对台外侨、防震减灾、地方志、保密、档案、气象、人防等工作。

　　强化社会治理。坚持法治与德治相结合，全面实施“七五”普法，深入推进精神文明建设，积极践行社会主义核心价值观，提高全民法治意识和道德自觉。创新基层社会治理，加强社区服务中心、社区服务站建设，建成社区公共服务综合信息平台，完成第十届村(居)民委员会换届选举工作。推进信访法治化、信息化建设，打造“阳光信访”。深化“平安乐山”建设，完善立体化、智能化、专业化社会治安防控体系，扎实推进防恐防暴、危爆物品、寄递物流、网络安全、禁毒防艾等集中整治，增强人民群众安全感。防范化解金融风险，依法打击非法集资。强化安全生产责任体系建设，提高重点行业、重点领域安全治理能力。完善应急管理体系，提高全民防范和应对突发事件能力。加强食品药品安全监管，打造“智慧食安”，申报创建国家级食品安全城市。加强国防和后备力量建设，促进军地互动发展、军民融合共建。

　　九、切实转变职能加强自身建设

　　坚持“干在实处、走在前列”工作取向，把抓落实作为政府工作的首要任务，不断增强政府系统执行力和公信力，努力建设群众满意的法治政府、创新政府、廉洁政府和服务型政府。

　　优化政务服务。认真落实法治政府建设实施纲要，加快法治政府建设。坚持简政放权、放管结合、优化服务，推进决策公开、执行公开、管理公开、服务公开和结果公开，营造事项最少、程序最简、办事最快、服务最优的发展环境。深入推进“互联网+政务服务”等信息惠民工程，探索推进政府公共数据资源开放和共享利用，打破政府“信息孤岛”。充分发挥新兴媒体作用，构建政务新媒体矩阵平台，扩大公众参与，主动回应社会关切。完善行政权力依法规范公开运行平台、网上移动办事大厅，加快建设公共资源电子交易平台。抓好乐山政务服务国家级标准化试点项目建设，开展相对集中行政许可权改革和综合行政执法工作试点，推行“一窗受理”模式，实现“双随机一公开”全覆盖。

强化执行落实。坚持把清单制、责任制作为推动落实的重要方法，围绕挂图作战项目、供给侧结构性改革、产业发展、脱贫攻坚、民生工程等重点工作，强化项目化、清单化、台账化管理，确保责任落实到人、进度明确到月，集中力量办成大事、办好实事、办妥难事。突出目标导向和问题导向，围绕“走在前列”下目标，对着问题开药方，促进中央、省市各项决策部署落地生根、取得实效。抓住想干事、敢干事这两个关键点，健全行政问责与激励机制，探索容错纠错机制，加强政务督查、效能监督和发展环境治理，不断凝聚干事创业、争创一流的强大合力。

　　巩固廉政防线。认真落实党风廉政建设“两个责任”，完善权力运行制约和监督机制，形成有权必有责、用权必担责、滥权必追责的制度安排。健全廉洁风险防控体系，推进廉洁风险迹象记分管理，实现重点领域、关键环节和重点岗位廉政监管全覆盖。严格执行中央八项规定和省市十项规定精神，规范政府债务管理，严格控制经费支出。始终保持反腐高压态势，以零容忍态度惩治腐败。主动接受市人大及其常委会法律监督、工作监督和市政协民主监督，广泛听取民主党派、无党派人士、工商联、人民团体和社会各界的意见，自觉接受社会监督。

　　各位代表：责任重于泰山，发展时不我待。让我们更加紧密地团结在以习近平同志为核心的党中央周围，在省委、省政府和市委的坚强领导下，紧紧依靠全市人民，保持定力、拼搏实干、锐意进取，奋力开创乐山转型发展新局面，以优异成绩迎接党的十九大和省第十一次党代会胜利召开!
